# Photon-counting CT versus energy-integrating detectors for cardiac imaging: a systematic review of evidence from in vivo human studies on image quality and radiation dose

**DOI:** 10.1186/s12880-025-01825-8

**Published:** 2025-07-25

**Authors:** Magnar Rønning, Elias Johansen, Albertina Rusandu

**Affiliations:** 1 Department of Circulation and Medical Imaging, University of Science and Technology, Trondheim, Norway; 2 Department of Radiology, Volda Hospital, Møre og Romsdal Hospital Trust, Volda, Norway

**Keywords:** Photon counting CT, Cardiac CT, Image quality, Radiation dose

## Abstract

**Background:**

Computed tomography (CT) is central to cardiovascular diagnostics, with coronary CT angiography (CCTA) widely used for evaluating coronary artery disease (CAD) due to its high sensitivity and negative predictive value. However, conventional energy-integrating detector (EID) CT is limited by reduced contrast resolution and artifacts, especially in patients with heavy calcification or stents, which can impair diagnostic accuracy. Photon-counting CT (PCCT) is an emerging technology that directly converts X-ray photons into electrical signals, offering improved spatial resolution, contrast-to-noise ratio (CNR), and dose efficiency. While phantom studies have demonstrated its potential, clinical validation remains limited. This systematic review assesses image quality and radiation dose of PCCT versus EID in human in vivo cardiovascular imaging studies.

**Methods:**

A systematic literature search was conducted in PubMed and Embase following PRISMA guidelines. Studies comparing PCCT and EID in cardiovascular imaging in human patients were included. Outcomes of interest were image quality parameters (CNR, SNR, artifacts), subjective image quality, diagnostic confidence, and radiation dose metrics.

**Results:**

Eleven studies met the inclusion criteria, encompassing a range of cardiovascular applications including CCTA, stent assessment, and coronary calcium scoring. Across studies, PCCT consistently demonstrated the potential to improve diagnostic image quality at similar radiation doses, or to maintain the image quality while enabling significant reductions in radiation dose and, in some cases, contrast media volume. Artifact reduction, especially for blooming around calcifications, was frequently reported. However, variation in imaging protocols and outcome measures limited direct comparisons.

**Conclusion:**

Current evidence from human in vivo studies supports that PCCT offers significant advantages in terms of image quality and radiation dose in cardiac CT examinations. Subjective image quality is particularly enhanced, while objective parameters remain influenced by technical factors and protocol selection. While findings are promising, especially for patients with complex coronary pathology, further large-scale, standardized studies are needed to confirm diagnostic and prognostic benefits in routine clinical practice.

**Supplementary Information:**

The online version contains supplementary material available at 10.1186/s12880-025-01825-8.

## Introduction

Computed tomography (CT) has significantly advanced medical care by providing high-resolution anatomical imaging, thereby improving diagnostic accuracy, reducing unnecessary procedures, and supporting more effective treatment planning. CT plays a vital role in the diagnosis and management of cardiovascular diseases, which remains the leading cause of death worldwide [1]. Coronary CT angiography (CCTA) is regarded as a first-line diagnostic tool for assessing coronary artery disease (CAD), owing to its high sensitivity and negative predictive value in patients at low to intermediate risk [2]. Furthermore, CT allows for assessing coronary stenosis and plaque composition, coronary artery calcification scoring (CACS), assessing stents or acquiring planning images for heart valve replacement (TAVI), offering important prognostic insights [3, 4]. However, it has some limitations, i.e., limited contrast resolution, suboptimal tissue characterization ability [5]. Diagnostic accuracy may be reduced in patients with extensive calcified atherosclerosis or previous stent placement. In these populations, imaging artifacts, such as beam hardening and partial volume effects (blooming artifact), can obscure the visualization of the vessel lumen and potentially result in an overestimation of stenosis severity [6, 7].

Photon-counting computed tomography (PCCT) employs a fundamentally different detector technology compared to conventional CT. Traditional energy-integrating detectors (EIDs) use a scintillator layer that absorbs incoming X-ray photons and converts their energy into visible light. This light is then detected by a semiconductor photodiode, which generates an electrical signal for image formation. In contrast, photon-counting detectors (PCDs) eliminate the scintillator layer. Instead, x-ray photons interact directly with a semiconductor material, where each photon is counted and its energy measured [8–10]. This yields significant advantages, including reduced image noise, enhanced contrast-to-noise ratio (CNR), and artifact reduction, thereby improving overall image quality and diagnostic confidence [8, 10].

By minimizing electronic noise and improving both contrast-to-noise ratio (CNR) and the visualization of small anatomical structures, PCCT demonstrates superior dose efficiency compared to EID [11] As a result, PCCT offers significant potential to enable effective diagnostic imaging at reduced radiation doses [10].

A multitude of cardiovascular phantom studies have been conducted on PCCT; however, the number of corresponding in vivo patient studies remains limited. To the best of our knowledge, this is the first study that systematically reviews the existing literature assessing image quality and radiation doses of cardiovascular imaging using PCCT vs. EID in human studies. This study aims to provide an overview of how the advantages of PCCT over EID, related to image quality and/or radiation dose reduction initially demonstrated in phantom studies, have been validated in clinical cardiac imaging studies.

## Methods

The systematic review was performed following PRISMA guidelines [12].

### Search strategy and selection process

The search was conducted in PubMed and Embase. The PubMed search included several keywords and MeSH terms. The same search strategy was used in Embase, by replacing the MeSH terms with corresponding definitions from Embase’s indexing system, Emtree. The search terms and the syntax are provided in Table 1. The Boolean operators “OR” and “AND” were used to combine search terms, while “NOT” was avoided to reduce further the chance of losing relevant articles. The searches were performed in May 2025.

**Table 1:** The search syntax

Concept	Search terms	PubMed	Embase
#1 PCCT	“Photon Counting Computed Tomography”	169	572
	“Photon Counting CT”	398	457
	“PCCT”	256	277
	“Spectral CT”	746	953
	**Total number of hits for PCCT**	1,277	1,719
#2 Dose	“Radiation Dosage”	90,640	128,979
	“Radiation Dose”	39,323	155,164
	“Dose Reduction”	13,071	82,827
	“Low Dose”	108,423	109,063
	“Effective Dose”	17,686	18,729
	“CTDI”	1,122	1,174
	**Total number of hits for Dose**	235,672	345,853
#3 Image quality	“Diagnostic Imaging”	3,030,652	236,260
	“Image Noise”	3,298	3,516
	“Spatial Resolution”	27,274	28,120
	“Contrast Resolution”	1,611	1,581
	“Artifact Reduction”	1,443	5,624
	“Low Contrast Detectability”	212	215
	**Total number of hits for Image Quality**	3,039,932	270,897
#4 Heart	“Coronary Angiography”	77,248	30,492
	“Cardiac CT”	2,652	2,959
	“Coronary CT Angiography”	2,426	2,556
	“CCTA”	2,916	3,016
	“Heart CT”	40	84
	**Total number of hits for Heart**	79,791	35,843
#Search strategy PubMed	((“Photon Counting Computed Tomography“[tw] OR “Photon Counting CT“[tw] OR “PCCT“[tw] OR “Spectral CT“[tw]) AND (“Radiation Dosage“[Mesh] OR “Radiation Dose“[tw] OR “Dose Reduction“[tw] OR “Low Dose“[tw] OR “Effective Dose“[tw] OR “CTDI“[tw]) AND (“Diagnostic Imaging“[Mesh] OR “Image Noise“[tw] OR “Spatial Resolution“[tw] OR “Contrast Resolution“[tw] OR “Artifact Reduction“[tw] OR “Low Contrast Detectability“[tw]) AND (“Coronary Angiography“[Mesh] OR “Cardiac CT“[tw] OR “Coronary CT Angiography“[tw] OR “CCTA“[tw] OR “Heart CT“[tw]))	19	
#Search strategy Embase	(‘photon counting computed tomography’ OR ‘photon counting ct’ OR ‘pcct’ OR ‘spectral ct’) AND (‘radiation dosage’/exp OR ‘radiation dose’ OR ‘dose reduction’ OR ‘low dose’ OR ‘effective dose’ OR ‘ctdi’) AND (‘diagnostic imaging’/exp OR ‘image noise’ OR ‘spatial resolution’ OR ‘contrast resolution’ OR ‘artifact reduction’ OR ‘low contrast detectability’) AND (‘coronary angiography’/exp OR ‘cardiac ct’ OR ‘coronary ct angiography’ OR ‘ccta’ OR ‘heart ct’) AND [medline]/lim		17

The selection process was guided by a list of inclusion and exclusion criteria (Table 2).

**Table 2:** Inclusion and exclusion criteria

Inclusion criteria	Exclusion criteria
Studies involving PCCT	Studies on animals
In-vivo studies on humans	Studies examining anatomical structures other than the heart.
Studies reporting original data available in English	Technical reports from vendors supplying PCCT technology or associated systems
Included in Medline	Case reports and review articles

After removing duplicates, articles were initially screened for potential relevance through examination of their titles and abstracts. Those meeting the initial criteria were then assessed in full text for eligibility based on the predefined criteria. Title, abstract, and full-text screening were conducted independently by three reviewers.

To minimize the risk of overlooking relevant studies, reference lists of included articles and excluded review articles were searched for additional articles of relevance. The further search resulted in two articles.

Bias assessemnet of the included studies was performed (Appendix) with a custom-made quality assessment tool previously used to analyse image quality related studies [13] based on a revised QUADAS-2 quality assessment tool [14].

### Data extraction

Data extraction was performed by three reviewers. Extracted data included study characteristics and aspects related to the research question (image quality, radiation doses). The study characteristics registered were year, country, type of study, patient population, quality assessment approaches, and number of observers. Image quality related data that were extracted were objective measurements of image quality (with focus on parameters as SNR, CNR, and artifacts) and subjective assessments of image quality. Regarding radiation dose, all relevant dose-related variables were recorded, including CTDIvol and effective dose. In addition, exposure parameters and use of contrast agents were registered to provide context for the results.

## Results

**Fig. 1:**
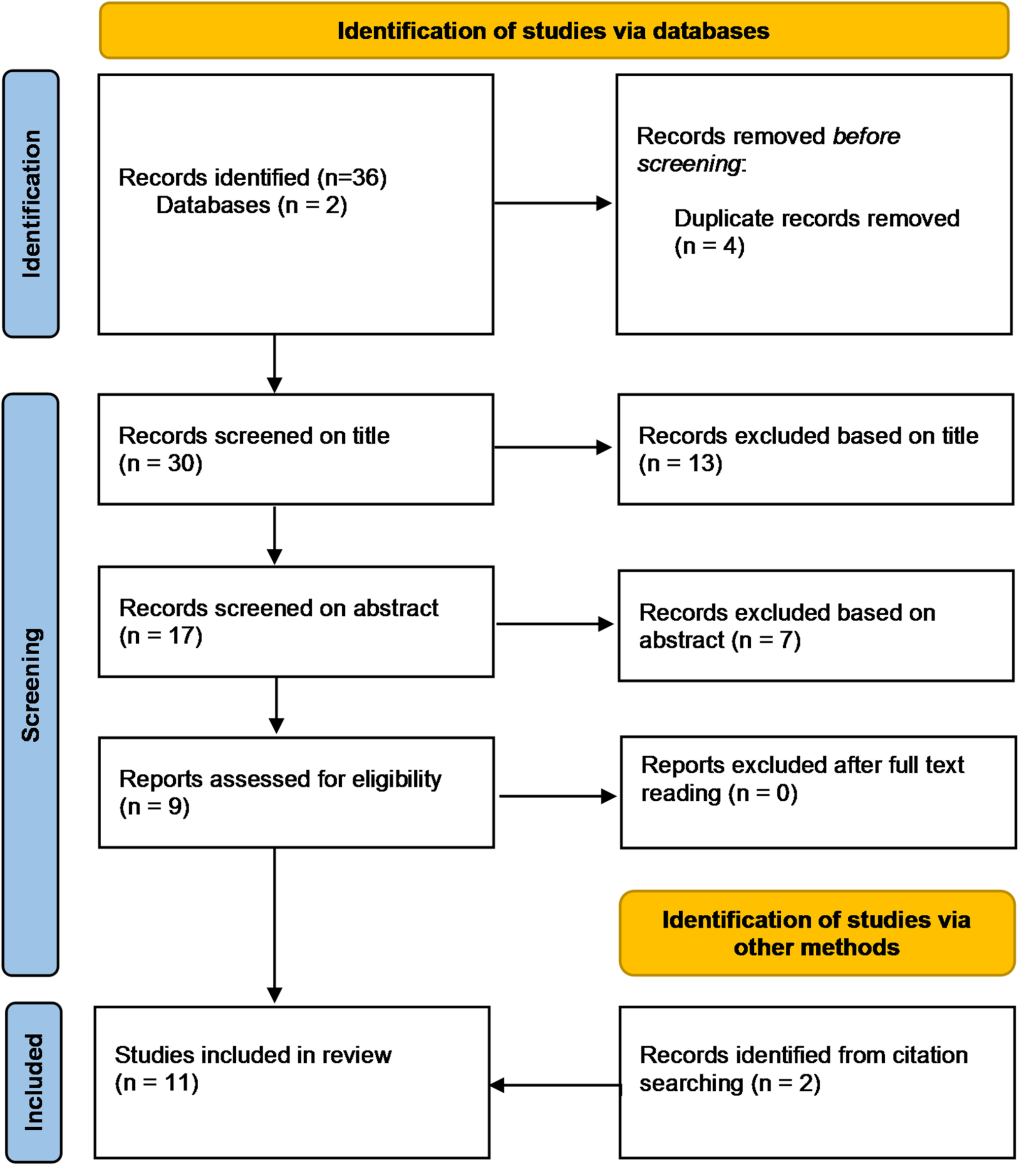
Prisma flow chart

The systematic search initially identified 36 articles (Table 1). Following the selection process, nine studies met the inclusion criteria. Additionally, two more studies were identified through citation searching, bringing the final number of included studies to eleven (Fig. 1).

**Table 3:** Overview of the included articles

Author and year	Study type	Aim	PCCT scanner used	Image quality assessment	Radiation dose parameters
Boccalini et al. [15] 2022	Prospective In vivo (*n* = 8)	To compare the image quality of stents assessment between PCCT and EID	SPCCT Prototype Philips	Blooming artifact Subjective image quality assessment	CTDIvol and DLP
Cundari et al. [16] 2024	Prospective In vivo (*n* = 100)	To evaluate the potential of contrast media reduction on PCCT	Dual source Naeotom Siemens	CNR Vascular attenuation Subjective image quality scoring	-
Dirrichs et al. [17] 2024	Retrospective In vivo (*n* = 300)	To compare image quality, suitability for TAVI and radiation dose on PCCT vs. EID	Naeotom Alpha Siemens	SNR CNR Visual image quality rating	CTDIvol, DLP and effective dose
Eberhard et al. [18] 2021	Prospective in vivo + phantom (*n* = 20)	To evaluate the accuracy of CAC score on PCCT vs. EID	Naeotom Alpha Siemens	image quality levels	CTDIvol, DLP and effective dose
Greffier et al. [19] 2023	Prospective in vivo +fantom (*n* = 8)	To evaluate the quality VMI from PCCT vs. EID-DECT	SPCCT Prototype Philips	NPS Detectability index Subjective noise, lumen conspicuity and sharpness, and overall image quality	CTDIvol and DLP
Haag et al. [20] 2024	Retrospective In vivo (*n* = 170)	To evaluate the accuracy of CACS on true non-contrast vs. virtual non-contrast on PCCT	Naeotom Alpha Siemens	CACS	DLP
Koons et al. [21] 2024	Prospective in vivo (*n* = 23)	Stenosis assessment on PCCT vs. EID	Dual source Naeotom Siemens	-Stenosis diameter -Blooming artefacts	CTDIvol
Schwartz et al. [22] 2023	Prospective In vivo (*n* = 10)	To compare the performance of CACS evaluation in PCCT vs. EID	Naeotom Alpha Siemens	SNR CNR CACS	CTDIvol and DLP
Si-Mohamed et al. [23] 2022	Prospective In vivo (*n* = 14)	To compare the image quality of and diagnostic confidence PCCT and EID	Prototype Philips	-NPS, CNR, SNR -detectability index -diagnostic quality score for coronary calcification, stent, and non-calcified plaque	CTDIvol snd DLP
Symons et al. [24] 2019	Prospective In vivo, ex vivo + phantom (*n* = 10)	To evaluate the performance of PCCT for CAC score compared to EID	Prototype- en EID replaced by a PCC detector in a dual source Somatom Definition Flash Siemens	CACs CNR	-
Van Der Bie et al. [25] 2024	Retrospective In vivo + phantom (*n* = 143)	Image quality and radiation dose in CTA on PCCT vs. EID	Naeotom Alpha Siemens	Detectability index Spatial resoution Image noise Iodine contrast	normalized CTDIvol

### Study characteristics

All included studies involved in vivo testing, with some also including phantom and/or ex vivo testing. Among these, eight were prospective studies and three were retrospective. The eleven studies collectively included a total of 800 patients, and the sample size varied between 8 and 300. Retrospective studies generally featured higher patient numbers compared to prospective studies. The studies assessed both image quality and radiation dose, except for two [16, 24] that focused solely on image quality. The studies included either subjective assessments of image quality, objective measurements of image quality parameters such as SNR and CNR, or a combination of both approaches. Table 3 summarizes the characteristics of each study, including details on the image quality assessment approach and measured parameters. The CT images were assessed independently by at least two radiologists who were blinded to details regarding scanner type and scanning and reconstruction parameters. The radiologists had at least four years’ experience, with many having over 20 years of clinical expertise.

**Table 4:** Key outcomes of the included studies

Author and year	Main findings	Image quality	Radiation dose
Boccalini et al. [15] 2022	Improved objective and subjective image quality on PCCT. This might overcome the current limits of CT for stent assessment	Significantly reduced blooming and higher subjective image quality scores on PCCT	Lower CTDIvol and DLP on PCCT (25,7 vs. 35,7mGy and 476 vs. 698 mGycm)
Cundari et al. [16] 2024	The increased image quality of PCCT allows reducing contrast media volume by 40%	Lower CNR with low contrast media dose but adequate vascular attenuation Similar subjective image quality scores	-
Dirrichs et al. [17] 2024	PCCT improves image quality for TAVI despite significantly reduced radiation dose	Lower CNR and SNR but higher visual image quality on PCCT	Lower mean CTDIvol, DLP and effective dose on PCCT (22,4 vs. 62,9mGy, 519 vs. 895 mGycm, 8,7 vs. 15,s mSv)
Eberhard et al. [18] 2021	Accurate CACS at lower radiation doses on PCCT vs. EID	Similar CACS	2.0 to 8,6mGy
Greffier et al. [19] 2023	PCCT improved spatial resolution, noise texture, noise magnitude, and detectability of the coronary lumen compared with DE- EID.	Higher contrast between the iodine and background material Higher subjective image quality scores	Not significantly different but slightly lower on PCCT
Haag et al. [20] 2024	Execellent correlation between CACS on true non-contrast virtual non-contrast images on PCCT	Similar CACS	Radiation dose reduction of 19.7%
Koons et al. [21] 2024	Clearer delineation of lumen and calcification improved the assessment of coronary artery stenosis with dense calcifications. It could spare patients from unnecessary interventional procedures.	Better overall image quality with PCCT. Blooming artifact decreased compared to EID	Mean CTDIvol 36 for PCCT vs. 55 for EID
Schwartz et al. [22] 2023	Comparable CACS accuracy	Slightly higher CNR (9.1vs 7,8) and quite similar SNR (3,1 vs. 3,2) on EID	Lower mean CTDIvol and DLP on PCCT (2,1 vs. 4,5 mGy and 38,7 vs. 78,4 mGycm)
Si-Mohamed et al. [23] 2022	Improved image quality and diagnostic confidence compared with EID	-No significant difference in noise -Reduced beam-hardening artifact on PCCT -Higher SNR and CNR on EID -Higher scores for overall image quality and diagnostic confidence with PCCT	significantly lower CTDIvol (25.7 mGy vs. 31.6 mGy) and DLP (411 mGy vs. 592 mGy) with PCCT than with EID
Symons et al. [24] 2019	PCCT has potential to improve CAC score image quality and/or to reduce radiation dose while maintaining diagnostic image quality.	Higher CNR with PCCT	-
Van Der Bie et al. [25] 2024	Patients can be scanned with lower kV with PCCT	Improved spatial resolution in PCCT Higher detectability indices in PCCT No reduction in iodine attenuation when using different kV Same noise levels, depending on the kernel used	Significantly lower CTDIvol comparing to EID

### CCTA

A small-scale prospective study [23] showed that PCCT outperformed EID dual-layer CT in improving image quality and diagnostic confidence of coronary CT angiography at a lower radiation dose. PCCT showed improvement in visualization of stents, coronary calcifications, coronary noncalcified plaque, coronary lumen, and coronary wall, together with better scores for subjective image noise, sharpness, and reduction of blooming artifacts (Table 4). However, CNR and SNR were higher on EID. Reduction of blooming artifacts was confirmed by Koons et al. [21], who also demonstrated better scores for subjective image quality and improved assessment of coronary artery stenosis in patients with dense calcifications at lower radiation doses. A study focused on stent assessment [15] also confirmed the significant reduction of blooming artifacts and higher subjective image quality scores on PCCT at lower radiation dose levels compared to EID, previously documented by phantom testing [26, 27].

A retrospective study analyzing PCCT images of different patient sizes demonstrated higher detectability indices compared to EID in large patients without increasing radiation dose [25]. However, the study showed that small and medium-sized patients, who are usually scanned with lower voltages on EID, require higher radiation doses to uphold image quality in high-resolution PCCT.

Two of the included articles address Virtual Monoenergetic Images (VMI) on PCCT. A study comparing VMIs from spectral PCCT and dual-energy (DE) EID [19] found that PCCT VMIs demonstrated superior spatial resolution, lower noise magnitude, and improved noise texture. The results showed better lumen conspicuity and sharpness, and overall subjective image quality scores at quite similar radiation dose levels. A study that aimed to determine the optimal energy level for VMI and assess the potential for reducing contrast agent usage in CCTA concluded that 45 keV was the most favorable level and that PCCT enables a reduction in contrast agent volume by 40% while maintaining diagnostic image quality [16].

The largest of the included studies [17] compared image quality and radiation dose in TAVI planning using PCCT versus DE-EID in 300 patients. Although objective image quality metrics such as CNR and SNR were lower with PCCT, subjective assessments by both radiologists and cardiologists consistently favored PCCT, with higher scores across multiple image quality parameters. Additionally, average radiation dose values were lower with PCCT, and the authors concluded that PCCT is better suited for TAVI planning than DE-EID.

### CACS

Three prospective studies assessed the accuracy of CACS on PCCT compared to EID. In addition to the similar CACS accuracy shown in all three studies [18, 22, 24], two of them documented significantly higher CNR on the PCCT images [22, 24]. All three studies demonstrated the positive impact of PCCT in CACS [18, 22, 24]. Symons et al. [24] evaluated the performance of PCCT compared to an EID system for CACS examinations, using both standard and low-dose protocols, and demonstrated superior performance of PCCT at low radiation doses compared to EID. Schwartz et al. [22] also demonstrated that PCCT may enable a considerable reduction in radiation dose while maintaining sufficient diagnostic image quality. The study performed by Eberhard et al. [18] showed an even greater dose reduction potential with PCCT with the same CACS accuracy at 2 mGy compared to 8,6 mGy on EID.

Haag et al. [20] assessed differences in CACS based on true non-contrast (TNC) images and virtual non-contrast (VNC) images derived from contrast-enhanced CCTA performed with PCCT. The results showed a strong correlation in Agatston scores between TNC and VNC. The authors concluded that VNC images have the potential to replace TNC scans that accounted for approximately 20% of the total radiation dose from CCTA.

## Discussion

This review summarizes the current evidence from in vivo human studies supporting the clinical advantages of PCCT over conventional EID systems in the context of cardiac imaging. Across multiple applications—including CCTA, CACS, stenosis and stent evaluation, and pre-procedural planning for TAVI—PCCT consistently demonstrated the potential to improve diagnostic image quality at similar radiation doses, or to maintain the image quality while enabling significant reductions in radiation dose and, in some cases, contrast media volume. Other potential advantages of PCCT, for instance, application in CT-derived fractional flow reserve, which, according to preclinical studies, enables non-invasive, lesion-specific evaluation of stenosis hemodynamic significance, an assessment that previously required invasive coronary angiography [1] have not yet been tested in human studies.

### Image quality and diagnostic confidence

The included studies consistently reported enhanced subjective image quality scores with PCCT compared to EID, despite mixed results in objective metrics such as CNR and SNR. For instance, in TAVI planning, Dirrichs et al. [17] reported lower CNR and SNR for PCCT; however, radiologists and cardiologists rated PCCT images higher in terms of diagnostic usefulness, ultimately concluding that PCCT was better suited for clinical decision-making. A possible explanation is that radiologists may prioritize technical image parameters differently depending on the clinical task [28], and in this context, spatial resolution could have been considered more important than other metrics such as CNR. Another factor that complicates the comparison of image quality between PCCT and EID is that identical scanning parameters are typically not used, as protocols are often optimized differently for each system based on their distinct technological capabilities. For instance, in the two studies that documented lower CNR with PCCT [17, 23], the ultra-high-resolution (UHR) scan mode was employed, which prioritizes spatial resolution over noise reduction. Sharper kernels and thinner slices contribute to a better spatial resolution, however, at the cost of increased noise and a reduced CNR [1]. Similarly, across multiple studies, including those by Boccalini et al. [15] and Si-Mohamed et al. [23], improvements in the visualization of stents, coronary lumen, and non-calcified plaque were noted, primarily due to reduced blooming artifacts and enhanced spatial resolution. A study evaluating different scan protocols found that in PCCT, the choice of reconstruction kernel primarily influences noncalcified coronary plaque quantification, while slice thickness predominantly affects calcified plaque measurements [29]. However, while subjective assessments strongly favor PCCT, the inconsistency in objective metrics like SNR and CNR highlights the need for further research into how quantitative image quality parameters relate to clinical outcomes.

### Radiation and contrast dose reduction

A major benefit of PCCT that has been demonstrated in a multitude of preclinical studies [30, 31] is its ability to maintain or enhance image quality while reducing patient exposure to ionizing radiation. This benefit was confirmed in the present review. Studies across a range of patient sizes and imaging protocols showed consistent reductions in CTDIvol and DLP, with the highest reported CTDIvol difference ranging from 2.0 to 8.6 mGy [18], corresponding to a radiation dose saving of approximately 77%. Haag et al. [20] point out that PCCT enables virtual non-contrast (VNC) reconstruction based on contrast-enhanced CCTA images, potentially eliminating the need for a separate true non-contrast (TNC) series. This approach could reduce the patient’s total radiation dose by approximately 19%. This represents a novel strategy for dose reduction, extending beyond traditional protocol adjustments. However, the literature recommends caution when using VNC images in routine clinical practice, even with PCCT, as small yet significant differences in attenuation values compared to TNC images may affect diagnostic accuracy [8].

In addition to radiation dose, PCCT also demonstrates potential in reducing contrast media requirements. Cundari et al. [16] found that PCCT allowed for a 40% reduction in contrast media volume in CCTA without compromising diagnostic quality, a finding of particular importance in patients with renal insufficiency or other contraindications to high iodine loads. Their study confirmed results from preclinical studies [32] and similar clinical studies of other organs, especially the aorta [33].

However, a key challenge remains the significant cost that limits the accessibility of this technology. In particular, high upfront costs and maintenance costs may restrict the broader implementation of PCCT in the near future [5]. Despite these high costs, PCCT may provide long-term savings by reducing contrast media usage, supporting multiple clinical applications in a single scan (for example, CACS and stenosis assessment), and potentially reducing the need for follow-up exams or additional imaging. However, clear evidence of cost-effectiveness is not yet available.

The current literature review has several limitations that are important to recognize. First, PCCT is still a relatively new technology that has only recently been introduced into clinical practice, and several of the included studies are based on prototype scanners with varying technical specifications. This makes it challenging to directly translate the results into widespread clinical use. The studies were conducted using different types of equipment and using various scanning protocols. This may have influenced the measurements and weakened the comparability of the findings. Furthermore, several of the included studies have small sample sizes, with five of them involving no more than 20 patients, and in certain studies, patients were excluded due to technical issues such as motion artifacts, which affect the results and further limit their generalizability.

## Conclusion

Although the number of in vivo PCCT studies focusing on the heart remains limited, the majority of these studies confirm findings previously established in phantom-based research such as PCCT’s capability to either improve diagnostic image quality at radiation doses similar to conventional EID or maintain image quality while significantly lowering radiation exposure volume in multiple cardiac imaging applications. However, larger multicenter trials with standardized protocols are warranted to confirm these findings and to define optimal PCCT imaging strategies across diverse patient populations.

## Electronic supplementary material

Below is the link to the electronic supplementary material.

Supplementary Material 1

## Data Availability

All data generated or analyzed during this study is provided within the manuscript.
