# Supplementary material for: Photon-counting CT versus energy-integrating detectors for cardiac imaging: a systematic review of evidence from in vivo human studies on image quality and radiation dose
Source: BMC Med Imaging. 2025 Jul 25;25:295. doi: 10.1186/s12880-025-01825-8 (PMC12297571; doi:10.1186/s12880-025-01825-8)
Supplement: Supplementary file 1 — Supplementary Material 1 [file 12880_2025_1825_MOESM1_ESM.docx]

**Appendix**

Bias assessment

| Author and year | Design | Patient selection | Image quality assessment | Nr. of observers | Inter-reader agreement | Score |
| --- | --- | --- | --- | --- | --- | --- |
| Boccalini et al (15) 2022 | Paired study  (n=8) | consecutive | independent  blinded | 3 | ? | 4/6 |
| Cundari et al (16) 2024 | Randomized  (n=100) | consecutive | independent  blinded | 2 | good to excellent | 6/6 |
| Dirrichs et al (17) 2024 | Retrospective non-paired non-randomized (n=300), last 100 with EID, first 200 with PCCT | consecutive | independent  blinded | 4 | moderate | 5/6 |
| Eberhard et al (18) 2021 | Paired study  (n=20) | consecutive | performed by software | N/A | N/A | 6/6 |
| Greffier et al (19) 2023 | Paired study  (n=8) | consecutive | in consensus  blinded | 2 | N/A | 4/6 |
| Haag et al (20) 2024 | Paired study  (n=170) | consecutive | performed by software | N/A | N/A | 6/6 |
| Koons et al (21) 2024 | Paired study  (n=23) | consecutive | performed by software | N/A | N/A | 6/6 |
| Schwartz et al (22) 2023 | Paired study  (n=10) | consecutive | performed by software | N/A | N/A | 5/6 |
| Si-Mohamed et al (23) 2022 | Paired study  (n=14) | consecutive | independent  blinded | 3 | slightly to moderate | 4/6 |
| Symons et al (24) 2019 | Paired study  (n=10) | consecutive | performed by software | N/A | N/A | 5/6 |
| Van Der Bie et al (25) 2024 | Retrospective non-paired non-randomized (n=143), last 96 with EID, first 47 with PCCT | consecutive | performed by software | N/A | N/A | 5/6 |
